# Cumulative High-Risk Pregnancy Complications and Stunting Risk in Indonesian Children Younger Than 5 Years: Retrospective Analysis Using the Developmental Origins of Health and Disease Framework

**DOI:** 10.2196/85742

**Published:** 2026-02-26

**Authors:** Widyawati Widyawati, Hafifah Maulia Ristandiati

**Affiliations:** 1 Department of Pediatric and Maternity Nursing, Faculty of Medicine, Public Health and Nursing, Universitas Gadjah Mada, Jl. Farmako, Sekip Utara, Kec. Depok, Kabupaten Sleman, Daerah Istimewa Yogyakarta, 55281, Indonesia, 62 274 545674; 2 Neonatal Intensive Care Unit, Universitas Gadjah Mada Academic Hospital, Universitas Gadjah Mada, Daerah Istimewa Yogyakarta, Indonesia

**Keywords:** pregnancy complications, maternal health, high-risk pregnancy, child nutrition disorders, stunting, Developmental Origins of Health and Disease

## Abstract

**Background:**

Stunting affects 21.6% of Indonesian children younger than 5 years, with complications from high-risk pregnancies (HRPs) identified as a potential risk factor. The Developmental Origins of Health and Disease framework suggests that prenatal exposures may permanently alter physiological development and disease susceptibility later in life.

**Objective:**

This study aimed to examine the cumulative effects of HRP complications on the risk of stunting in Indonesian children younger than 5 years while controlling for socioeconomic confounders.

**Methods:**

A retrospective study was conducted in Sleman Regency, Indonesia, analyzing 450 children (300 children with stunting and 150 children without stunting) aged 12 to 59 months. Data were collected from maternal medical records, maternal and child health handbooks, and integrated health post reports. Multivariate logistic regression was used to adjust for socioeconomic confounders including maternal education, family income, and antenatal care (ANC) visits.

**Results:**

Mothers of children with stunting had significantly higher rates of any HRP complications (206/300, 68.7% vs 48/150, 32%; *P*<.001). After adjustment, multiple HRP complications (≥2 conditions) showed the strongest association with stunting (adjusted odds ratio [aOR] 5.80, 95% CI 3.26‐10.32), exceeding the risk associated with individual complications such as anemia (aOR 3.21, 95% CI 2.12‐4.86) or preeclampsia (aOR 4.37, 95% CI 2.18‐8.76). Maternal education (aOR 0.72, 95% CI 0.58‐0.89), family income (aOR 0.68, 95% CI 0.52‐0.89), and ANC visits (aOR 0.85, 95% CI 0.76‐0.95) were identified as protective factors.

**Conclusions:**

The dose-response relationship between cumulative HRP complications and stunting supports the Developmental Origins of Health and Disease hypothesis. Current ANC protocols emphasizing single risk factors may be insufficient. Integrated prenatal care addressing cumulative risks is essential for stunting prevention in Indonesia.

## Introduction

### Overview

Stunting, defined as height-for-age *z* score ≤−2 SDs below the World Health Organization (WHO) Child Growth Standards median [1], represents a chronic nutritional disorder reflecting failures in health, nutrition, and psychosocial care [2]. Globally, an estimated 149 million children younger than 5 years were stunted in 2023, with the burden disproportionately concentrated in low- and middle-income countries (LMICs) [3]. Indonesia reports one of Southeast Asia’s highest stunting prevalence rates at 21.6% according to the 2023 National Nutritional Status Survey [4], though this masks significant regional disparities ranging from 7.2% in Bali to 39.4% in Central Papua [5]. Beyond short stature, stunting leads to irreversible cognitive deficits [6], reduced educational attainment, lower adult productivity, and intergenerational poverty perpetuation [7]. This original research article aims to examine the cumulative effects of high-risk pregnancy (HRP) complications on the risk of stunting in Indonesian children younger than 5 years, applying the Developmental Origins of Health and Disease (DOHaD) framework.

The DOHaD hypothesis provides a critical framework for understanding stunting etiology. First, proposed by Barker (1990) and later expanded by Hanson and Gluckman (2015), this theory posits that environmental exposures during sensitive developmental windows have permanent effects. Recent reviews have increasingly emphasized that these effects are often the result of cumulative and synergistic exposures, rather than single insults [8], which forms the central hypothesis of our study. These exposures can alter physiological structure, metabolic programming, and disease susceptibility later in life [9,10]. Central to this concept is fetal programming, where prenatal insults such as those from HRP complications disrupt normal development [11]. In LMICs like Indonesia, where 48.9% of pregnant women experience anemia [12], the interplay between maternal nutritional status, HRP complications, and child growth outcomes represents a critical pathway [13].

HRP complications, including anemia, preeclampsia, gestational diabetes mellitus (GDM), heart disease, and asthma, expose fetuses to nutrient deprivation, hypoxia, oxidative stress, and inflammation [14]. Maternal nutrition plays a critical role in fetal development, with deficiencies in key nutrients including iron, folate, and protein contributing to both HRP complications and subsequent child stunting [15]. Recent evidence suggests these effects may be cumulative, with multiple HRP complications synergistically increasing stunting risk beyond individual conditions [16-18]. For instance, a mother with both anemia and preeclampsia may expose the fetus to combined insults that disrupt fetal programming more severely than either condition alone [19]. However, most research examines HRP complications in isolation, with limited studies assessing their combined effects on stunting in Indonesia [20].

Despite Indonesia’s National Stunting Acceleration Strategy (2023‐2024), which emphasizes integrated nutrition interventions and antenatal care (ANC) strengthening, stunting prevalence remains above the WHO’s significance threshold (>20%) [21]. Current ANC protocols primarily focus on identifying single risks such as anemia or preeclampsia [22], potentially overlooking pregnancies with overlapping complications that confer exponentially higher risk [23]. Furthermore, the role of socioeconomic confounders such as maternal education, family income, and health care access requires careful consideration to clarify the independent association between HRP and stunting [24].

This study addresses these gaps by doing the following:

1. Analyzing the comprehensive association between HRP history and stunting in Indonesian children younger than 5 years using contemporary international guidelines [22,25]
2. Controlling for key socioeconomic and health care–related confounders
3. Applying the DOHaD framework to interpret findings within Indonesia’s public health context
4. Providing evidence for integrated prenatal care approaches that address cumulative risks rather than isolated complications.

### Key Messages

The key messages for this study are as follows:

1. Cumulative maternal complications during pregnancy significantly increase the risk of stunting among children younger than 5 years.
2. Integrating maternal health monitoring into early child nutrition programs may help prevent intergenerational undernutrition.
3. Findings support the DOHaD framework, emphasizing the importance of prenatal care quality for child growth.
4. Strengthening ANC screening for HRPs could be an effective policy to reduce stunting prevalence in Indonesia.

## Methods

### Study Design

A retrospective analytical study was conducted in Sleman Regency, Yogyakarta Special Region, Indonesia, between January 2019 and December 2024. The design followed the life course epidemiology framework and employed the counterfactual framework of causation [26]. The methodology adhered to the STROBE (Strengthening the Reporting of Observational Studies in Epidemiology) guidelines [27].

### Setting

Sleman Regency had 2272 documented stunting cases in 2024. The study focused on 6 subdistricts (Sleman, Brebah, Mlati, Kalasan, Godean, and Tempel), accounting for 91.6% of the regency’s stunting cases. Data were collected from the following:

1. Maternal medical records from public health centers
2. Maternal and child health (MCH) handbooks
3. Monthly integrated health post reports

### Participants

The study population consisted of children aged 12 to 59 months registered in the district Integrated Nutrition Information System in 2024.

### Eligibility Criteria

Inclusion criteria were as follows:

- Age 12‐59 months
- Complete MCH handbook with documented pregnancy history
- Complete medical records from prenatal care
- Complete anthropometric data

Exclusion criteria were as follows:

- Congenital anomalies
- Chronic diseases affecting growth
- Incomplete pregnancy history data
- Missing anthropometric measurements

### Sampling Strategy

A multistage cluster sampling method was used to obtain a representative sample of children aged 12 to 59 months in Sleman Regency. The process involved three stages.

#### Stage 1: Stratification of Subdistricts

Subdistricts were stratified based on the documented number of stunting cases:

- High burden: >400 stunting cases
- Medium burden: 200‐400 stunting cases
- Low burden: <200 stunting cases

#### Stage 2: Random Selection of Subdistricts

Two subdistricts were randomly selected from each stratum:

- High: Sleman (634 cases) and Berbah (445 cases)
- Medium: Mlati (400 cases) and Kalasan (271 cases)
- Low: Godean (196 cases) and Tempel (135 cases)

#### Stage 3: Probability Proportional to Size Sampling

For the final stage, we established a comprehensive sampling frame consisting of all 150 active integrated health posts within the 6 selected subdistricts, based on the registry from the Sleman District Health Office. From this list, we used probability proportional to size sampling to randomly select 33 integrated health posts, where the probability of selection was proportional to the number of children aged 12 to 59 months registered at each integrated health post.

Within each of the 33 selected integrated health posts, a systematic random sampling technique was used to recruit participants. The monthly attendance register of children aged 12 to 59 months served as the sampling frame. We calculated a fixed sampling interval (k) and aimed to recruit approximately 30 eligible children from each integrated health post until the target initial sample size of 990 children was achieved (33 integrated health post×30 children/integrated health post≈990). This approach ensured that the final sample was proportional to the population distribution across the selected subdistricts and that the recruitment process was both systematic and random.

### Participant Flow

A total of 990 eligible respondents were recruited from 6 subdistricts and 33 integrated health posts. However, due to incomplete data, 540 respondents were excluded. The final analytical sample comprised 450 children (300 children with stunting and 150 children without stunting).

### Sample Size Calculation

Using the formula for estimating a proportion in a finite population (N=62,817, Z=1.96, p=0.50, e=0.046), a total sample size of 450 children was determined [28].

### Variables

#### Dependent Variable

Stunting (height-for-age *z* score≤–2 SD; WHO 2006) is the dependent variable of the study.

#### Independent Variable

HRP complications (with timing of assessment) is the independent variable, including:

- Anemia: Diagnosed based on hemoglobin levels (Hb<11 g/dL) recorded during the first trimester (≤13 wk) and/or third trimester (≥28 wk) of pregnancy as documented in the MCH handbook, following WHO guidelines [22].
- Preeclampsia: Identified by new-onset hypertension (BP≥140/90 mmHg) with proteinuria occurring after 20 weeks of gestation as per routine ANC monitoring records, in line with American College of Obstetricians and Gynecologists guidelines [25].
- GDM: Diagnosed based on an abnormal oral glucose tolerance test (OGTT) result conducted between 24 and 28 weeks of gestation, using the International Association of Diabetes and Pregnancy Study Groups criteria [29].
- Heart disease: Documented preexisting or pregnancy-induced cardiac conditions diagnosed at any point during pregnancy and recorded in medical records, as defined by standard obstetric practice [25].
- Asthma: Physician-diagnosed asthma with exacerbations recorded during the pregnancy period in the MCH handbook or medical records, following the Global Initiative for Asthma report [30].
- HRP categorized as none, single, and multiple (≥2).

#### Confounding Variables

Maternal age, education, family income, ANC visits, iron consumption, maternal nutrition, and family size

#### Maternal Nutrition (Operational Definition)

Maternal nutritional status was assessed using prepregnancy or first-trimester BMI documented in the MCH handbook. BMI was calculated as weight in kilograms divided by height in meters squared (kg/m^2^). For this study, “inadequate maternal nutrition” was defined as a BMI less than 18.5 kg/m^2^ (underweight), as this represents a state of chronic undernutrition strongly linked to adverse pregnancy outcomes, following WHO classifications [31].

### Data Collection and Quality Assurance

Data were collected through a rigorous three-step process to ensure validity and minimize bias: (1) medical record review for HRP history and maternal variables, (2) MCH handbook review for detailed pregnancy history and ANC visits, and (3) direct verification of anthropometric data at integrated health posts.

### Quality Control and Measurement Validity

To ensure the highest data quality, a comprehensive quality assurance protocol was implemented.

- Training and standardization: All field staff responsible for data extraction and anthropometric measurements underwent a 3-day intensive training workshop prior to data collection. This included standardization exercises for both data abstraction and height measurement techniques, as recommended by the Demographics and Health Surveys Phase 8 Anthropometry Manual [32]. An inter-rater reliability assessment was conducted, showing high agreement (κ=0.89). A refresher training session was also held midway through the data collection period to prevent observer drift.
- Anthropometric measurement protocol: A child’s height was measured using a Seca 213 microtoise (precision: 0.1 cm). Two measurements were taken and averaged if the difference was less than 0.5 cm. All procedures strictly followed WHO protocols [1].
- Instrument calibration: The Seca 213 microtoise was calibrated for accuracy daily before the first measurement using a standard calibrated measuring rod by the field supervisor. Any instrument deviating by more than 0.1 cm was immediately replaced, a critical step for ensuring measurement validity [33].

### Statistical Analysis

Data were analyzed using Python (pandas 1.5.3, numpy 1.24.3, scipy 1.10.1; Integrated Development Environment). Descriptive statistics were presented as means with SD for continuous variables and frequencies with percentages for categorical variables. Bivariate analyses, including *χ*^2^ tests for categorical variables and *t* tests for continuous variables, were conducted to examine the unadjusted associations between independent variables and stunting, reporting unadjusted odds ratios (OR) with 95% CI. A multivariate logistic regression model was developed to determine the independent association between HRP complications and stunting while controlling for confounders. All identified confounding variables (maternal age, education, family income, ANC visits, iron tablet consumption, maternal nutrition, and family size) were entered into the model simultaneously using a forced entry method. The model fit was assessed using the Hosmer-Lemeshow goodness-of-fit test, Nagelkerke *R*^2^, and the area under the receiver operating characteristic curve. A 2-tailed *P* value less than .05 was considered statistically significant.

### Ethical Considerations

Ethical approval was obtained from the Ethics Committee Faculty of Medicine, Public Health, and Nursing, Universitas Gadjah Mada (Ref: KE/FK/0825/EC/2025). All data were anonymized to protect respondents’ privacy and confidentiality. Since this study was using retrospective data, informed consent was not needed. The study was conducted in accordance with the Declaration of Helsinki [34] and relevant bioethical principles [35].

## Results

### Participant Flow

**Figure 1.**
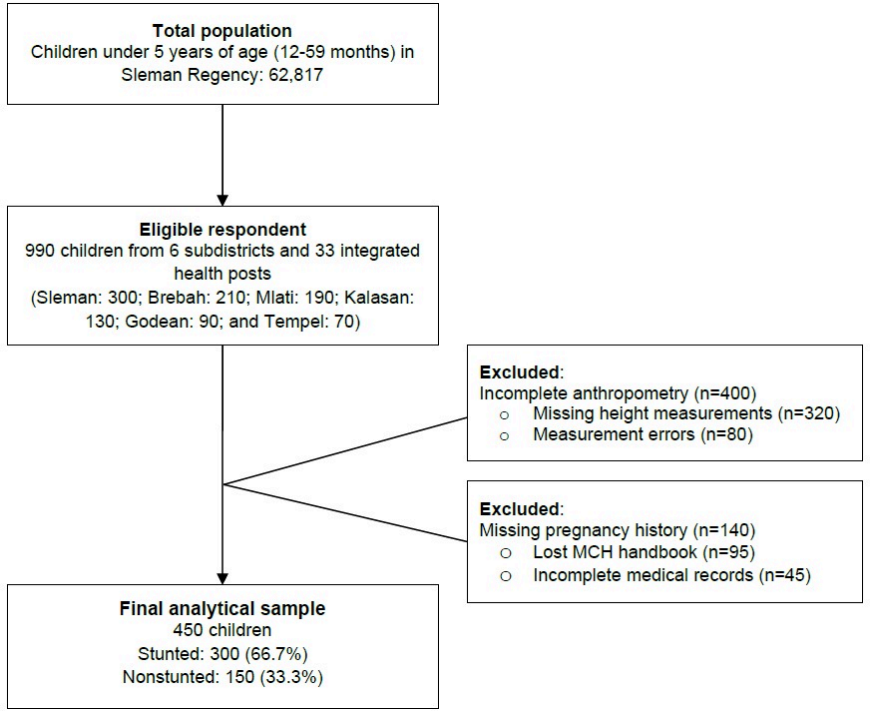
Flow diagram of participant selection in Sleman Regency, Indonesia. MCH: maternal and child health.

The participant selection process is detailed in Figure 1. From an initial pool of 990 children recruited from the selected integrated health posts, a total of 540 children were excluded due to incomplete data. The primary reasons for exclusion were incomplete anthropometric measurements (n=400) and incomplete pregnancy history data (n=140), which were treated as mutually exclusive categories. This resulted in a final analytical sample of 450 children (300 children with stunting and 150 without stunting).

### Characteristics of Study Participants

The final sample of 450 children was drawn from 6 subdistricts, with the number of participants from each stratum being proportional to the documented stunting cases (high: Sleman and Berbah; medium: Mlati and Kalasan; and low: Godean and Tempel). The distribution of children with and without stunting across these subdistricts was comparable (*P*=.87). The characteristics of the study participants are presented in Table 1.

**Table 1.** Comparison of child and maternal characteristics between children with stunting (n=300) and those without stunting (n=150) in Sleman Regency, Indonesia.

Variable and category	Children with stunting (n=300)	Children without stunting (n=150)	*P* value
Child characteristics			
Age (mo), mean (SD)	34.8 (12.9)	34.1 (12.6)	.43
Sex, n (%)			.68
Female	142 (47.3)	74 (49.3)	
Male	158 (52.7)	76 (50.7)	
Maternal characteristics			
Education, n (%)			<.001
No education	15 (5.0)	3 (2.0)	
Elementary school	35 (11.7)	8 (5.3)	
Junior high school	85 (28.3)	18 (12.0)	
Senior high school	149 (49.7)	99 (66.0)	
College/university	16 (5.3)	22 (14.7)	
Family income (IDR^a^), n (%)			<.001
Low (<1.5 million)	187 (62.3)	52 (34.7)	
Medium (1.5‐3 million)	89 (29.7)	68 (45.3)	
High (>3 million)	24 (8.0)	30 (20.0)	
ANC^b^ visits, n (%)			<.001
<6 visits	98 (32.7)	21 (14.0)	
≥6 visits	202 (67.3)	129 (86.0)	
Iron tablets, n (%)			<.001
<90 tablets	176 (58.7)	48 (32.0)	
≥90 tablets	124 (41.3)	102 (68.0)	
Maternal nutrition, n (%)			<.001
Inadequate	193 (64.3)	43 (28.7)	
Adequate	107 (35.7)	107 (71.3)	
Family size, n (%)			<.001
≤4 members	98 (32.7)	89 (59.3)	
>4 members	202 (67.3)	61 (40.7)	

a US $1=16,740.00 IDR.

b ANC: antenatal care.

Table 1 presents participant characteristics and the sampling process. The mean age was 34.6 (SD 12.8) months, with no significant difference between stunted (34.8, SD 12.9 mo) and nonstunted (34.1, SD 12.6 mo) groups (*P*=.43). Gender distribution was similar (158/300, 52.7% male in stunted group vs 76/150, 50.7% in nonstunted group; *P*=.68). Significant differences were observed in maternal education (*P*<.001), family income (*P*<.001), ANC visits (*P*<.001), iron supplementation (*P*<.001), maternal nutrition (*P*<.001), and family size (*P*<.001).

### Prevalence of HRP Complications

Table 2 shows the prevalence of HRP complications. Any HRP complication was significantly higher among mothers of children with stunting (206/300, 68.7% vs 48/150, 32%; *P*<.001). Anemia was the most common complication (145/300, 48.3% vs 34/150, 22.7%, *P*<.001), followed by preeclampsia (46/300, 15.3% vs 6/150, 4%; *P*<.001). A clear dose-response relationship was observed: children exposed to multiple HRP complications had 6.71 times higher odds of stunting (OR 6.71, 95% CI 3.54‐12.72).

**Table 2.** Prevalence of individual and cumulative high-risk pregnancy (HRP) complications among mothers of children with and without stunting, with unadjusted odds ratios (ORs) for stunting.

HRP component	Children with stunting (n=300)	Children without stunting (n=150)	Unadjusted OR (95% CI)	*P* value
Any HRP, n (%)	206 (68.7)	48 (32.0)	4.67 (3.10‐7.04)	<.001
Individual complications, n (%)				
Anemia (Hb^a^<11 g/dL)	145 (48.3)	34 (22.7)	3.17 (2.10‐4.78)	<.001
Preeclampsia	46 (15.3)	6 (4.0)	4.33 (1.83‐10.24)	<.001
Gestational diabetes	28 (9.3)	7 (4.7)	2.07 (1.01‐4.25)	.05
Heart disease	12 (4.0)	2 (1.3)	3.08 (0.68‐13.94)	.09
Asthma	18 (6.0)	5 (3.3)	1.86 (0.69‐5.01)	.16
Number of HRP complications, n (%)				
None	94 (31.3)	102 (68.0)	Reference	—^b^
One complication	132 (44)	36 (24.0)	3.98 (2.49‐6.36)	<.001
Two or more complications	74 (24.7)	12 (8.0)	6.71 (3.54‐12.72)	<.001

a Hb: hemoglobin.

b Not applicable.

### Bivariate and Multivariate Analysis

**Figure 2.**
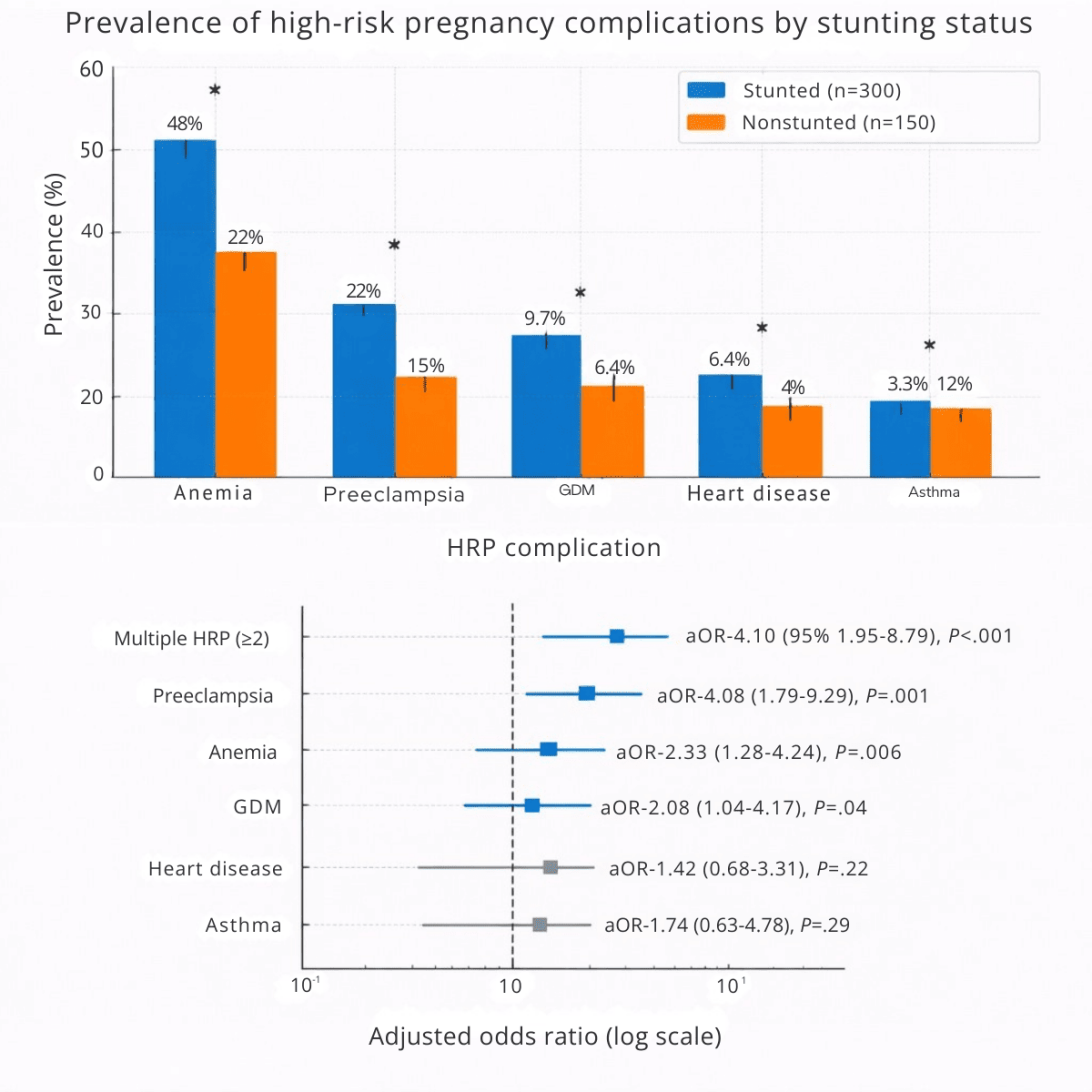
Adjusted odds ratios (aOR) from multivariate logistic regression showing the association between high-risk pregnancy (HRP) complications and stunting. Error bars represent 95% CIs. GDM: gestational diabetes mellitus.

Table 3 presents bivariate and multivariate analysis results. After adjustment for all confounders, anemia (adjusted OR [aOR] 3.21, 95% CI 2.12‐4.86), preeclampsia (aOR 4.37, 95% CI 2.18‐8.76), and gestational diabetes (aOR 2.85, 95% CI 1.42‐5.72) remained significantly associated with stunting. Children exposed to multiple HRP complications showed a 5.8-fold increased risk (aOR 5.80, 95% CI 3.26‐10.32). Among confounders, maternal education (aOR 0.72, 95% CI 0.58‐0.89), family income (aOR 0.68, 95% CI 0.52‐0.89), and ANC visits (aOR 0.85, 95% CI 0.76‐0.95) were independently protective (Figure 2).

**Table 3.** Bivariate and multivariate logistic regression analysis of factors associated with stunting in children aged 12‐59 months (n=450)^a^.

Variable	Unadjusted OR^b^ (95% CI)	*P* value	aOR^c^ (95% CI)	*P* value
HRP^d^ complications				
Anemia (Hb^e^<11 g/dL)	3.17 (2.10‐4.78)	<.001	3.21 (2.12‐4.86)	<.001
Preeclampsia	4.33 (1.83‐10.24)	<.001	4.37 (2.18‐8.76)	<.001
Gestational diabetes	2.07 (1.01‐4.25)	.05	2.85 (1.42‐5.72)	.003
Heart disease	3.08 (0.68‐13.94)	.09	2.63 (0.57‐12.14)	.22
Asthma	1.86 (0.69‐5.01)	.16	1.74 (0.63‐4.79)	.29
Number of HRP complications				
None	Reference	—^f^	Reference	—
One complication	3.98 (2.49‐6.36)	<.001	3.45 (2.14‐5.56)	<.001
Two or more complications	6.71 (3.54‐12.72)	<.001	5.80 (3.26‐10.32)	<.001
Confounding factors				
Maternal age (per year increase)	0.98 (0.94‐1.02)	.34	1.01 (0.96‐1.06)	.73
Maternal education (per level increase)	0.65 (0.52‐0.81)	<.001	0.72 (0.58‐0.89)	.003
Family income (per category increase)	0.58 (0.45‐0.75)	<.001	0.68 (0.52‐0.89)	.005
ANC^g^ visits (per visit increase)	0.79 (0.71‐0.88)	<.001	0.85 (0.76‐0.95)	.006
Iron tablet consumption (<90 vs ≥90)	3.01 (2.03‐4.47)	<.001	1.21 (0.75‐1.95)	.43
Maternal nutrition (inadequate vs adequate)	4.47 (2.98‐6.71)	<.001	1.34 (0.81‐2.21)	.25
Family size (per person increase)	1.42 (1.23‐1.64)	<.001	1.08 (0.92‐1.27)	.34

a Model fit statistics: Hosmer-Lemeshow test: *χ*2=6.84, *P*=.55; Nagelkerke *R*2=0.412; area under receiver operating characteristic curve 0.812. Key finding: Multiple HRP complications (≥2) showed the strongest association with stunting (aOR 5.80), exceeding individual risks like anemia (aOR 3.21) or preeclampsia (aOR 4.37).

b OR: odds ratio.

c aOR: adjusted odds ratio.

d HRP: high-risk pregnancy.

e Hb: hemoglobin.

f Not applicable.

g ANC: antenatal care.

### Model Fit Statistics

The multivariate model showed good fit (Hosmer-Lemeshow test: *χ*^2^=6.84, *P*=.55; Nagelkerke *R*^2^=0.412; area under receiver operating characteristic curve=0.812) with no multicollinearity (all variance inflation factor <2.5).

## Discussion

### Main Findings and Theoretical Implications

This study provides compelling evidence that the cumulative burden of HRP complications, rather than isolated conditions, is the most powerful prenatal factor associated with stunting in Indonesian children. Our finding of a dose-response relationship, where exposure to two or more HRP complications increased stunting risk nearly 6-fold (aOR 5.80), offers strong empirical support for the DOHaD hypothesis [16]. Critically, our findings extend the DOHaD framework by demonstrating that it is not merely the presence of a single prenatal insult but the synergistic interaction of multiple simultaneous exposures that most profoundly disrupts fetal programming and elevates disease risk later in life [10,11,16]. This challenges the current ANC paradigm in Indonesia, which predominantly focuses on identifying and managing single risks like anemia or preeclampsia, potentially overlooking pregnancies with overlapping complications that confer substantially higher risk.

While anemia and preeclampsia remained significant independent predictors, their impact must be contextualized within the broader landscape of cumulative risk. Anemia (aOR 3.21) was the most prevalent single complication in our study (48.3% in the stunted group), consistent with Indonesia’s high national maternal anemia burden of 48.9% [12]. However, its effect size was substantially lower than that of multiple HRP exposures. Similarly, preeclampsia (aOR 4.37), while strongly associated, was also outpaced by the combined risk. These findings indicate that isolated management of single risks during ANC may miss HRPs with overlapping complications, underscoring the need to understand the synergistic biological pathways that make the cumulative effect so profound.

### Biological Mechanisms of Cumulative Risk

Our finding that multiple HRP complications exponentially increase stunting risk suggests synergistic biological effects rather than a simple additive model. According to the DOHaD framework, the fetus adapts to the intrauterine environment. However, multiple simultaneous stressors can overwhelm these adaptive mechanisms, leading to permanent alterations in developmental programming. Several interconnected pathways likely explain this phenomenon. First, placental dysfunction serves as a central hub. Conditions like preeclampsia directly impair placental blood flow, a process increasingly understood through specific placental biomarkers that predict adverse outcomes [36], leading to fetal hypoxia and nutrient restriction. When combined with maternal anemia, which reduces the oxygen-carrying capacity of the blood, the placenta’s ability to deliver oxygen and nutrients is severely compromised, amplifying fetal growth restriction [19]. Second, oxidative stress and inflammation are common final pathways for many HRP complications. Preeclampsia, GDM (through hyperglycemia), and anemia (through hypoxia-reperfusion injury) all independently increase the production of reactive oxygen species. The concurrent presence of these conditions creates a pro-inflammatory and prooxidant intrauterine environment that can damage developing fetal tissues and disrupt metabolic programming [37]. Finally, the combined metabolic disruption is critical. For instance, a fetus exposed to both maternal anemia (nutrient deprivation) and GDM (excess glucose) receives conflicting and damaging signals, forcing it to adapt to both scarcity and toxicity simultaneously. This metabolic dissonance can have a more profound impact on endocrine and cardiovascular system development than either condition alone, setting the stage for postnatal growth faltering and stunting [38].

### Role of Socioeconomic and Health Care Factors

While the biological mechanisms explain the heightened vulnerability from cumulative HRP exposures, our analysis also identified key protective factors. Maternal education (aOR 0.72), family income (aOR 0.68), and ANC visits (aOR 0.85) were significantly associated with reduced stunting risk. However, their modest effect sizes reveal a complex reality and highlight the limitations of current interventions. This complexity is further illustrated by the shifting significance of iron tablet consumption and maternal nutrition. Both showed strong associations in bivariate analysis but lost significance after adjustment for socioeconomic confounders, suggesting their effects are largely mediated by upstream factors like education and income [26]. It appears that mothers with higher education and greater resources are better equipped to maintain adequate nutrition and adhere to supplementation, positioning these socioeconomic conditions as more fundamental determinants of child growth [3,14]. This finding underscores that interventions focusing solely on nutrient provision or increasing ANC visit frequency, without addressing the underlying socioeconomic context, are likely to have limited long-term impact on stunting prevention.

### Strengths

This study has several methodological strengths. First, the large, representative sample with rigorous multistage cluster sampling enhances external validity. Second, comprehensive adjustment for socioeconomic and health care confounders strengthens our analytical approach. Third, the use of contemporary international guidelines [22,25] for HRP diagnosis ensures methodological rigor and comparability. Fourth, triangulation of multiple data sources minimizes misclassification bias. Fifth, the application of advanced statistical methods with appropriate model fit assessment ensures the robustness of our findings.

### Limitations

Despite these strengths, several limitations must be acknowledged. Our interpretation of the findings is constrained by the study’s retrospective observational design and data sources, which also impact the generalizability of our results.

- Study design and causality: The retrospective, cross-sectional nature of our analysis limits our ability to establish temporality and infer direct causality. While we have identified strong and statistically significant associations, we cannot confirm that the observed HRP complications directly caused stunting, only that they are robustly linked within the DOHaD framework.
- Potential for selection bias: A significant limitation is the exclusion of 540 participants (54.5% of the initial sample) due to incomplete data. This raises a substantial risk of selection bias. It is plausible that mothers and children excluded from the analysis faced greater socioeconomic challenges or more severe health complications, which could lead to an overestimation of the effect sizes reported. Future research should implement more robust data collection strategies to minimize exclusions and ensure findings are more generalizable.
- Data constraints and unmeasured mediators: Our reliance on secondary data from medical records and MCH handbooks, while practical, is subject to potential inaccuracies in documentation. Furthermore, we could not assess the severity or gestational timing of HRP complications, which may have differential impacts. Critically, our dataset did not include key perinatal and postnatal mediators such as birth weight, gestational age, and breastfeeding practices that are central to the DOHaD framework. Consequently, we are unable to quantify the indirect effects of HRP and can only report the total effect, not the specific direct and indirect pathways. For instance, birth weight and gestational age are critical outcomes of a complicated pregnancy and are themselves strong predictors of stunting [7]. Similarly, breastfeeding practices (eg, exclusivity and duration) can be influenced by maternal health and are a major determinant of postnatal growth [7]. Future research should aim to integrate these perinatal and postnatal variables to provide a more complete understanding of the DOHaD mechanisms in this context [9].
- Generalizability of findings: This study was conducted in Sleman Regency, a region with relatively high development indices and health care access compared to many high-burden areas in Indonesia, such as Central Papua [5,39]. Therefore, while the fundamental biological relationship between cumulative HRP and stunting is likely universal [10], the specific prevalence and effect sizes observed may not be directly generalizable to less-resourced settings. Our findings are most directly applicable to similar regencies in Java, and they serve as a crucial call for further research in more diverse contexts.

### Implications for Policy and Practice

Our findings align with studies from other LMICs showing strong associations between maternal HRP and child stunting. A recent systematic review by Beal et al [20] reported similar effect sizes for anemia and preeclampsia in Indonesian children. However, our study adds significant value by being the first to examine multiple HRP complications simultaneously, demonstrating their synergistic cumulative effect while controlling for a comprehensive set of confounders using contemporary international guidelines [22,25].

The Indonesian context presents unique challenges and variations. In our study area of Sleman, anemia prevalence was high (48.3%), yet the national stunting burden shows stark disparities, from 7.2% in Bali to 39.4% in Central Papua [4,5], a pattern where the latest analyses confirm that socioeconomic factors remain a primary driver of stunting nationally [13,40]. This complex landscape, characterized by a dual burden of malnutrition and epidemiological transition, underscores why a single-risk approach is insufficient. Our analysis demonstrates that cumulative risk assessment better predicts stunting in this diverse environment.

Given this robust evidence, our findings have direct and critical implications for MCH policy in Indonesia. The current single-risk-focused ANC paradigm is insufficient to address the complex, cumulative risks we have identified. We recommend the following actionable shifts:

- Revise ANC protocols to incorporate cumulative risk assessment: National guidelines should be updated to include a cumulative risk scoring system. Pregnancies with two or more identified HRP complications should be automatically flagged as “very high risk” and prioritized for intensive, integrated management involving both obstetricians and nutritionists.
- Integrate MCH services: To break the intergenerational cycle of malnutrition, health information systems must be linked. A mother’s history of cumulative HRP complications should trigger targeted postnatal follow-up for her infant, including enhanced growth monitoring and nutritional support, regardless of the infant’s initial anthropometric status.
- Strengthen health system capacity: Addressing cumulative risk requires a well-trained and empowered workforce. Investment in training for ANC providers to identify, manage, and counsel patients with multiple complications is essential. This includes training on the DOHaD framework to enhance their understanding of the long-term implications of prenatal care quality.

Strengthen health system Capacity: Addressing cumulative risk requires a well-trained and empowered workforce. Investment in training for ANC providers to identify, manage, and counsel patients with multiple complications is essential. This includes training on the DOHaD framework to enhance their understanding of the long-term implications of prenatal care quality.

By adopting a cumulative risk framework aligned with the DOHaD hypothesis, Indonesia can accelerate progress toward reducing stunting and breaking the cycle of intergenerational malnutrition.

### Research Recommendations

Prospective cohort studies are needed to establish temporality and explore mediating pathways in the HRP-stunting relationship. Implementation research on integrated HRP-stunting prevention programs could identify effective delivery strategies. Finally, exploration of biological mechanisms could advance our understanding of developmental programming and identify novel intervention targets.

### Conclusion

In conclusion, this study provides compelling evidence that the cumulative burden of HRP complications is the most powerful factor associated with stunting in Indonesian children. While individual complications remain significant, their impact is substantially lower than that of cumulative exposures. This challenges the current single-risk-focused ANC paradigm and highlights the urgent need for a shift toward integrated, cumulative risk management strategies. Such a paradigm shift, aligned with the DOHaD framework, is essential for effectively breaking the intergenerational cycle of malnutrition in Indonesia.

## Abbreviations

ANC: antenatal care
aOR: adjusted odds ratio
DOHaD: Developmental Origins of Health and Disease
GDM: gestational diabetes mellitus
HRP: high-risk pregnancy
LMICs: low- and middle-income countries
MCH: maternal and child health
OR: odds ratio
STROBE: Strengthening the Reporting of Observational Studies in Epidemiology
WHO: World Health Organization

## References

[1] Child growth standards. World Health Organization.

[2] Prendergast AJ, Humphrey JH (2014). The stunting syndrome in developing countries. Paediatr Int Child Health.

[3] (2023). Levels and trends in child malnutrition: UNICEF/WHO/World Bank group joint child malnutrition estimates: key findings of the 2023 edition. https://iris.who.int/server/api/core/bitstreams/ccd825e2-e6d0-4101-bedd-8189355dcd81/content.

[4] (2025). SSGI 2024: national stunting prevalence drops to 19,8%. BKPK Ministry of Health: Health Development Policy Agency.

[5] (2023). Stunting in Indonesia and its determinants. Badan Kebijakan Pembangunan Kesehatan.

[6] Dewey KG, Begum K (2011). Long-term consequences of stunting in early life. Matern Child Nutr.

[7] Victora CG, Adair L, Fall C (2008). Maternal and child undernutrition: consequences for adult health and human capital. Lancet.

[8] Sal-Sarria S, González-Pardo H, Conejo NM (2025). Synergistic impact of early-life stress and prenatal immune activation on spatial memory and oxidative metabolism in rat cortico-limbic networks. Neurobiol Learn Mem.

[9] Hanson MA, Gluckman PD (2015). Developmental origins of health and disease-global public health implications. Best Pract Res Clin Obstet Gynaecol.

[10] Gluckman PD, Hanson MA, Cooper C, Thornburg KL (2008). Effect of in utero and early-life conditions on adult health and disease. N Engl J Med.

[11] Burton GJ, Jauniaux E (2018). Pathophysiology of placental-derived fetal growth restriction. Am J Obstet Gynecol.

[12] (2023). Indonesian health survey (SKI) 2023. BKPK Ministry of Health: Health Development Policy Agency.

[13] Atamou L, Rahmadiyah DC, Hassan H, Setiawan A (2023). Analysis of the determinants of stunting among children aged below five years in stunting locus villages in Indonesia. Healthcare (Basel).

[14] Siramaneerat I, Astutik E, Agushybana F, Bhumkittipich P, Lamprom W (2024). Examining determinants of stunting in urban and rural Indonesian: a multilevel analysis using the population-based Indonesian family life survey (IFLS). BMC Public Health.

[15] Nadhiroh SR, Micheala F, Tung SEH, Kustiawan TC (2023). Association between maternal anemia and stunting in infants and children aged 0-60 months: a systematic literature review. Nutrition.

[16] Evans GW, Li D, Whipple SS (2013). Cumulative risk and child development. Psychol Bull.

[17] Greve LT, Fentz HN, Trillingsgaard T (2024). Cumulative risk predicting differential effectiveness of the incredible years parent-training. J Appl Dev Psychol.

[18] Appleyard K, Egeland B, van Dulmen MHM, Sroufe LA (2005). When more is not better: the role of cumulative risk in child behavior outcomes. J Child Psychol Psychiatry.

[19] Buciu VB, Ciurescu S, Șerban DM (2025). The compounded risk of maternal anemia and preeclampsia: neonatal outcomes and predictive modeling in a low-resource tertiary center. J Clin Med.

[20] Beal T, Tumilowicz A, Sutrisna A, Izwardy D, Neufeld LM (2018). A review of child stunting determinants in Indonesia. Matern Child Nutr.

[21] (2018). National strategy to accelerate stunting prevention 2018-2024. https://www.globalfinancingfacility.org/sites/default/files/Indonesia-GFF-Investment-Case-ENG.pdf.

[22] (2023). Anaemia in women and children. World Health Organization.

[23] Ghodsi D, Omidvar N, Nikooyeh B, Roustaee R, Shakibazadeh E, Al-Jawaldeh A (2021). Effectiveness of community nutrition-specific interventions on improving malnutrition of children under 5 years of age in the eastern mediterranean region: a systematic review and meta-analysis. Int J Environ Res Public Health.

[24] Shenoy S, Sharma P, Rao A (2023). Evidence-based interventions to reduce maternal malnutrition in low and middle-income countries: a systematic review. Front Health Serv.

[25] (2019). Chronic hypertension in pregnancy. American College of Obstetricians and Gynecologists.

[26] Hernán MA, Robins JM (2020). Causal Inference: What If.

[27] (2023). STROBE Statement.

[28] Kish L (1965). Survey Sampling.

[29] Metzger BE, Gabbe SG, Persson B, International Association of Diabetes and Pregnancy Study Groups Consensus Panel (2010). International Association of Diabetes and Pregnancy Study Groups recommendations on the diagnosis and classification of hyperglycemia in pregnancy. Diabetes Care.

[30] (2024). Global strategy for asthma management and prevention. Global Initiative for Asthma.

[31] Phelps NH, Singleton RK, Zhou B (2024). Worldwide trends in underweight and obesity from 1990 to 2022: a pooled analysis of 3663 population-representative studies with 222 million children, adolescents, and adults. The Lancet.

[32] Croft TN, Allen CK, Zachary BW (2023). Guide to DHS statistics. The DHS Program.

[33] Cogill B (2001). Anthropometric indicators measurement guide. ULBI Repository.

[34] World Medical Association (2013). Declaration of Helsinki: ethical principles for medical research involving human subjects. JAMA.

[35] Beauchamp TL, Childress JF (2019). Principles of Biomedical Ethics.

[36] Hong J, Kumar S (2023). Circulating biomarkers associated with placental dysfunction and their utility for predicting fetal growth restriction. Clin Sci (Lond).

[37] Assani AD, Boldeanu L, Siloși I (2025). Pregnancy under pressure: oxidative stress as a common thread in maternal disorders. Life (Basel).

[38] Yajnik CS (2024). Early life origins of the epidemic of the double burden of malnutrition: life can only be understood backwards. Lancet Reg Health Southeast Asia.

[39] (2023). Renstra: Rencana Strategis 2023-2026. https://rsprespira.jogjaprov.go.id/wp-content/uploads/2024/09/Cetak-Renstra-Dinas-Kesehatan-DIY-2023-2026_blur-dikompresi.pdf.

[40] Arief YS, Yunita FC, Efendi F, Murti FAK, Pradipta RO, McKenna L (2025). Social and environmental determinants of childhood stunting in Indonesia: national cross-sectional study. JMIR Pediatr Parent.

